# *In Vitro* Characterization of Circulating Endothelial Progenitor Cells Isolated from Patients with Acute Coronary Syndrome

**DOI:** 10.1371/journal.pone.0056377

**Published:** 2013-02-11

**Authors:** Diana Campioni, Giorgio Zauli, Stefania Gambetti, Gianluca Campo, Antonio Cuneo, Roberto Ferrari, Paola Secchiero

**Affiliations:** 1 Department of Medical Sciences, Section of Hematology, Azienda Ospedaliero-Universitaria, Arcispedale Sant’Anna, University of Ferrara, Ferrara, Italy; 2 Institute for Maternal and Child Health, IRCCS Burlo Garofolo, Trieste, Italy; 3 Department of Medical Sciences, Cardiovascular Section, Azienda Ospedaliero-Universitaria, Arcispedale Sant’Anna, University of Ferrara, Ferrara, Italy; 4 Department of Morphology and Embryology and LTTA Centre, University of Ferrara, Ferrara, Italy; University of Sao Paulo – USP, Brazil

## Abstract

**Background:**

The current understanding of the functional characteristics of circulating endothelial progenitor cells (EPC) is limited, especially in patients affected by cardiovascular diseases. In this study, we have analyzed the *in vitro* clonogenic capacity of circulating EPC, also known as endothelial colony-forming cells (ECFC), in patients with acute coronary syndrome (ACS), in comparison to the colony forming unit-endothelial-like cells (CFU-EC) of hematopoietic/monocytic origin.

**Methodology/Principal Findings:**

By culturing peripheral blood mononuclear cells (PBMC) of patients with ACS (n = 70), CFU-EC were frequently isolated (from 77% of ACS patients), while EPC/ECFC were obtained only in a small subset (13%) of PBMC samples, all harvested between 7–14 days after the acute cardiovascular event. Notably, *ex-vivo* generation of EPC/ECFC was correlated to a higher *in vitro* release of PDGF-AA by the corresponding ACS patient PBMC. By using specific endothelial culture media, EPC/ECFC displayed *in vitro* expansion capacity, allowing the phenotypic and functional characterization of the cells. Indeed, after expansion, EPC/ECFC exhibited a normal diploid chromosomal setting by FISH analysis and an immunophenotype characterized by: *i*) uniform positivity for the expression of CD105, CD31, CD146 and Factor VIII, *i*) variable expression of the CD34, CD106 and CD184 markers, and *iii*) negativity for CD45, CD90, CD117 and CD133. Of interest, in single-cell replanting assays EPC/ECFC exhibited clonogenic expansion capacity, forming secondary colonies characterized by variable proliferation capacities.

**Conclusion/Significance:**

Our data indicate that a careful characterization of true EPC is needed in order to design future studies in the clinical autologous setting of patients with ACS.

## Introduction

Endothelial progenitor cells (EPC) have been described as a rare population of non-hematopoietic cells, which reside in the bone marrow, supporting the integrity of vascular endothelium [1]–[3]. These cells can be mobilized to the circulation under the effect of different chemokines and soluble angiogenic factors [4], [5]. In the early onset (within 4 hours after in-hospital admission) after myocardial infarction and after other types of major vascular injuries, spontaneous or induced mobilization of circulating hematopoietic stem cells, of colony forming unit-endothelial-like cells (CFU-EC) of hematopoietic/monocytic origin and of EPC have been described [6]–[13]. However, in spite of the phenotypic characterization of circulating EPC as being CD34^+^, CD133^+^, VEGFR-2^+^ and CD45^-^, there has been an immunophenotypic and morphological overlap between haematopoietic/monocytic colonies (CFU-EC) and EPC in the scientific literature. Such overlap has been often a source of misunderstanding [14]–[21]. Hence, the clonogenic properties of true EPC, also known as endothelial progenitor cells/endothelial colony-forming cells (ECFC), are incompletely defined. Only few studies have investigated the *in vitro* culture characteristics of the human circulating EPC/ECFC, which were identified in the early phase of myocardial infarction at the time of in-hospital admission [9]–[11]. On this basis, starting from the seminal studies of Ingram and Yoder [22], [23], who proposed a redefinition of the endothelial progenitor cells via clonal analysis, in the present study we have adopted a combined strategy of multiparametric flow cytometric analysis and of single cell replanting assays to establish the *in vitro* clonogenic expansion properties of circulating EPC, in patients with acute coronary syndrome (ACS).

For this purpose, we have assessed PBMC samples obtained by 70 acute patients with ACS diagnosis, by evaluating the functional and phenotypic characteristics of circulating EPC/ECFC.

## Materials and Methods

### Patients

The study population analysed in the present study included 70 patients admitted to the Coronary Care Unit with the diagnosis of ACS. According current guidelines, ACS was defined combining the following three criteria: *i*) typical chest pain lasting for ≥20 minutes in the last 24 hours; *ii*) ST-segment depression ≥0.5 mm or ST-segment elevation ≥1 mm in 2 or more contiguous peripheral; *iii*) elevation of myocardial necrosis markers (CK-MB and troponin I) above the normal range (5 and 0.15 ng/ml, respectively) in two or more separate blood samples. In addition, we analysed 18 patients admitted to our cardiology unit for rhythm disorder (15 third grade atrio-ventricular block, 3 Mobitz II atrio-ventricular block, 1 sinus-atrial block) undergoing definitive pace-maker implantation. Exclusion criteria were: presence of any known neoplastic disease, diseases affecting the immune system and ongoing infectious diseases. All patients were treated with medical therapy according current guidelines including aspirin, clopidogrel, heparin, ACE inhibitors, ß-blockers, statins and diuretics. At entry, patients were randomly allocated to receive ambulatory blood withdrawal, for evaluation of circulating EPC, at different intervals: days 3–6 and 7–14 after the acute event. The study was approved by the local ethic committee (Azienda Ospedaliero-Universitaria, Arcispedale Sant’Anna, University of Ferrara) and all participants provided written informed consent and all clinical investigations have been conducted according to the principles expressed in the according to the Helsinki declaration of human rights.

### Flow cytometric analysis of putative circulating EPC

A four-color cytometry analysis of whole fresh peripheral blood (PB) samples was performed, as previously described [24], on a FACSCalibur equipped with the four-color option (Becton Dickinson, San Diego, CA), in order to enumerate the circulating EPC (CD34^+^/CD133^+^/VEGFR-2^+^/CD45^-^ cells). Appropriate gate analysis was used for the detection of EPC excluding events of different origin, such as non-hematopoietic circulating cells and non-specifically stained events. At least 200.000 events were analyzed for each sample. The following antibodies (Ab) were used for FACS analysis: Flk-1/VEGF-R2 rabbit polyclonal IgG (Santa Cruz Biotechnology; Santa Cruz, CA) followed by anti-rabbit FITC Ab (DAKO, Milan, Italy); CD133 Ab (AC-133 PE; Miltenyi Biotech, Auburn, CA), CD45 Ab (2D1 APC or 2D1PercP; BD Biosciences Pharmingen,) and CD34 Ab (Q-Bend/10 PercP or Q-Bend/10 APC; BD Biosciences Pharmingen).

### Cytokines assay

Peripheral blood mononuclear cell (PBMC) suspensions were isolated by density-gradient centrifugation with lymphocyte cell separation medium (Cedarlane Laboratories, Hornby, Ontario, Canada). Patient PBMC were then seeded at a cell density of 2.5×10^6^/ml in serum free Iscove medium (Gibco BRL, Grand Island, NY) supplemented with 1% penicillin/streptomycin and 2% L-glutamine. After 48 hours of culture at 37°C in 5% CO_2_, PBMC conditioned supernatants were collected by centrifugation, followed by filtration through 0,22μm Millipore sterile filters (Millipore Corporation, Bedford, MA, USA), and froze at -20 °C. Samples were assessed in duplicate for the determination of angiogenic cytokine levels by using a searchlight human angiogenesis array 3-multiplex assay (Aushon Biosystems, Billerica, MA). Sensitivity of the assay was: 3.7 pg/ml for HB-EGF, 1.95 pg/ml for KGF and PDGF-AA, 11.7 pg/ml for TPO, 21.5 pg/ml for VEGFR-1 and 27.3 pg/ml for VEGFR-2.

### Short-term primary colony assay in liquid culture medium

In order to obtain *in vitro* EPC colonies, 3×10^6^ PBMC were seeded into Collagen I petri dishes (35 mm, Biocoat, BD Labware, Bedford, MA) by using three different culture media: *i)* M199 Glutamax I (Gibco BRL), supplemented with 20% of FCS, 1% penicillin/streptomycin and 1% L-glutamine; *ii*) long-term Medium 5100 (Voden, Milano, Italy), supplemented with 12.5% FBS, 12.5% HS, 1% L-glutamine, 1% penicillin-streptomycin; *iii*) EGM2 medium (Lonza, Walkersville, MD) with 2% FBS and full supplements (EGM2 Bullet kit, Lonza). Cultures were performed in triplicate and the detection of adherent colonies (id, aggregates with more than 50 cells) was monitored and scored as either EPC/ECFC or CFU-EC on the basis of morphological features, as previously described [6], [25]–[27].

### Flow cytometric analysis of cultured EPC/ECFC and CFU-EC

In order to analyze the immunophenotypic pattern of primary EPC/ECFC and CFU-EC, cells were detached with trypsin/EDTA (Gibco, BRL, UK) before the specific staining for flow cytometric analysis with the following Ab: CD45 Ab (2D1-APC), CD31 Ab (WM59-FITC), CD184 Ab (12G5-PE), CD105 Ab (SN6-PE), CD14 Ab (MΦP9-PE), CD146 Ab (P1H12-PE) (all purchased from BD Biosciences Pharmingen), CD34 Ab (QBend/10-PercP; Serotec Ltd., Oxford) and CD133 Ab (AC133-PE; Miltenyi). Gate on viable cells was defined upon staining with 7-amino-actinomycin D (7-AAD; BD Biosciences Pharmingen).

### Fluorescence in situ hybridization (FISH)

FISH analysis was carried out using an enumeration probe (i.e. Centromeric Probe CEP6 and CEP9) for a ploidy chromosome set evaluation. The probes were chosen from a commercially available list “Vysis FISH Chromosome probes” provided by Abbott Molecular (Abbott Park, IL). The FISH procedure was performed according to the manufacturer’s protocol. For each probe, 100 cells were evaluated. The cells were viewed through an epifluorescent microscope equipped with 100X oil objective lens and triple bandpass filter for DAPI, SpectrumGreen and SpectrumOrange.

### Immunocytochemical analysis

Immunohistochemistry analysis was carried out by performing the alkaline phosphatase anti-alkaline-phosphatase (APAAP) staining, as previously described [26], [27], using the following monoclonal Ab: Factor VIII Ab (F8/86; DAKO), CD31 (WM-59; BD), VEGF receptor-2 (KDR-1; Sigma-Chemical, St. Louis, MO), CD105 (8E11; Cymbus Biotechnology); CD106 (1G1b1; Valter Occhiena, Torino, Italy), CD14 (MΦP9; BD), and CD45 (HI30; BD). EPC/ECFC were identified on the basis of their positivity for: Factor VIII, CD31, VEGFR-2, CD105, CD106, and negativity for CD14 and CD45 markers.

### Single cell clonogenic assay

When primary colonies reached a size with more than 100 cells/colony, adherent cells were detached by using trypsin/EDTA (Gibco BRL), re-suspended in complete EGM-2 medium, and sub-cultured by seeding no more then 1 cell/well in a collagen I 96-well tissue culture dishes. Medium was changed every 3 days. Individual wells were daily examined under the microscope to score the number of cells growing per well. Subclones were classified on the basis of their *in vitro* expansion and progeny capacity, in agreement with the study of Barrandon and Green (1987) [28], as: holoclones, characterized by a high growth capacity; paraclones, characterized by cells with a short replicative lifespan; meroclones, considered as an intermediate stage.

### Statistical analyses

For each set of experiments, values were analysed by calculating medians, means±SD and box plots were used to show the median, minimum and maximum values, and 25th to 75th percentiles. The results were evaluated by using analysis of variance with subsequent comparisons by Student’s t-test and with the Mann-Whitney rank-sum test. Correlations between data were estimated using Spearman’s correlation coefficient. Statistical significance was defined as p<0.05.

## Results

### Phenotypic analysis of circulating CD34^+^/CD133^+^/VEGFR-1^+^/CD45^-^ cells in ACS patients

PB samples were obtained from a total of 70 ACS patients, with a mean age of 64.5±10.5 years, and a prevalence of male (72%). Patient main characteristics are reported in **Table S1**. Blood withdrawal was carried out at different intervals (up to 14 days) after the hospital admission for the acute cardiovascular event. The presence of the circulating CD34^+^/CD133^+^/VEGFR-1^+^/CD45^-^ cells, which are thought to correspond to EPC, was monitored by multi-parametric flow cytometry on fresh PB samples. Of note, the level of circulating CD34^+^/CD133^+^/VEGFR-2^+^/CD45^-^ cells in ACS patients was very low at any time point investigated (mean±SD: 0.017±0.013% with respect to total peripheral blood mononuclear cells, or 2.2±4.5 cells/µl of blood).

Of note, the levels of circulating CD34^+^/CD133^+^/VEGFR-2^+^/CD45^-^ cells in ACS patients were not significantly different with respect to the levels (mean±SD: 0.017±0.016% or 2.1±4.0 cells/µl of blood) measured in a group of 18 non-ACS patients (matched to the ACS patients for age and gender) admitted to our cardiology unit for rhythm disorder (15 third grade atrio-ventricular block, 3 Mobitz II atrio-ventricular block, 1 sinus-atrial block) undergoing definitive pace-maker implantation.

### Characterization of the clonogenic potential of PBMC derived from ACS patients

**Figure 1:**
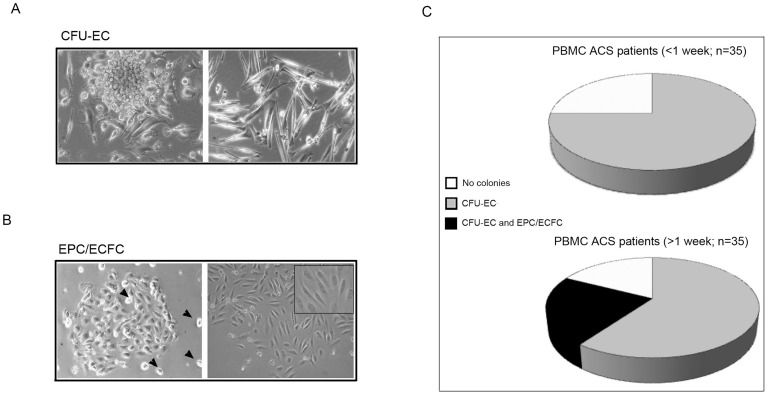
Characterization of the clonogenic potential of PBMC derived from ACS patients. PBMC samples obtained from ACS patients (n = 70) were seeded in collagen I coated wells for short-term primary colony assay in liquid culture medium. Cultures were monitored for 15 days for the presence of adherent colonies, scored on the basis of morphological features as: CFU-EC (**A,** left panel) or EPC/ECFC (**B,** left panel; arrowheads: hemopoietic mononucleated cells). In **A**, the right panel shows a monolayer of spindle-shaped endothelial-like monocytes. In **B**, the right panel shows a representative image of CFU-EC after *in vitro* expansion. Original magnification: 20X and 25X for the inset. In **C**, frequency of detection of CFU-EC and EPC/ECFC in PBMC of ACS patients, divided on the basis of the time of blood withdrawal after the hospital admission for the acute cardiovascular events.

PBMC samples obtained from the ACS patients were seeded in collagen I coated wells for short-term primary colony assay in liquid culture medium. Cultures were scored up to 15 days of culture for the presence and the morphology of adherent colony forming units of monocytes (CFU-EC; **Figure 1A**) and endothelial (EPC/ECFC; **Figure 1B**) origin. CFU-EC colonies, as previously described [6], [24], were characterized by a central cluster of endothelial-like monocytic cells (**Figure 1A**), sometimes forming also tubular structures. CFU-EC could be frequently (77%) derived from the ACS patients, irrespectively of time of blood withdrawal (**Figure 1C**). Of note, CFU-EC did not displayed *in vitro* expansion capacity and their endothelial differentiation resulted defective, in spite of using different endothelial specific media supplemented of pro-angiogenic cytokines.

Primary EPC/ECFC appeared as a small cluster of cells growing within the *in vitro* adherent cell fraction mainly composed by temporary adherent hemopoietic mononucleated cells (**Figure 1B,** left panel). Differently from CFU-EC, EPC/ECFC could be derived from the PBMC of a limited subset of the ACS patients (9/70) all harvested at late time points (between 7–14 days) after acute cardiovascular events (**Figure 1C**). Of interest, EPC/ECFC when kept in culture became a larger monolayer (**Figure 1B,** right panel), thus exhibiting *in vitro* expansion capacity, differently from CFU-EC.

### Analysis of pro-angiogenic cytokines release by PBMC

**Figure 2:**
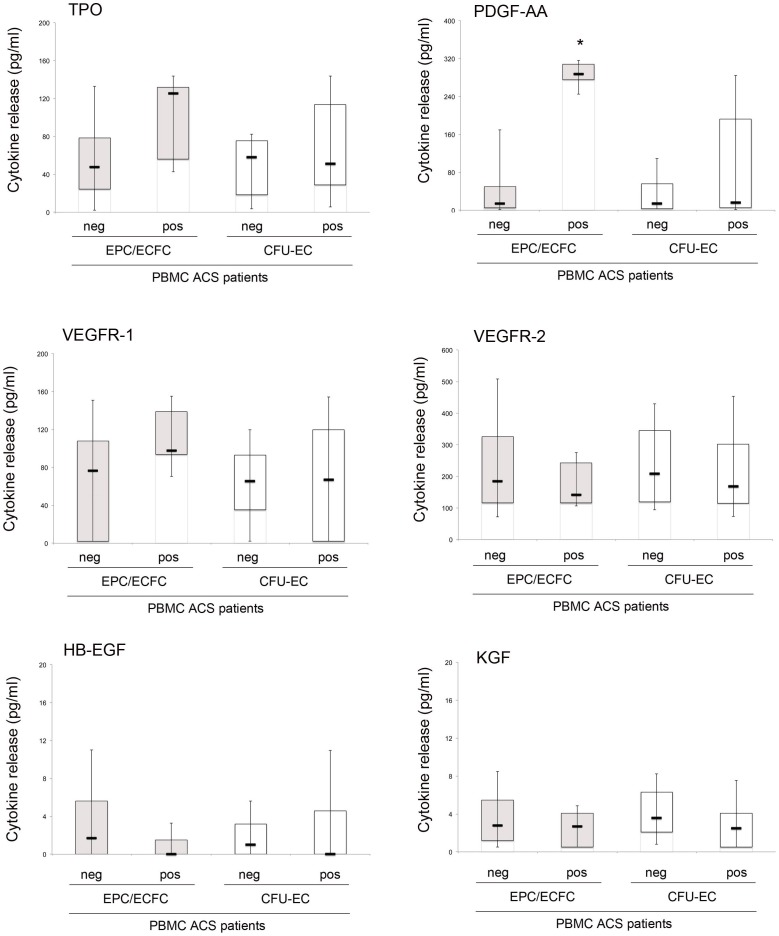
Analysis of pro-angiogenic cytokines release by PBMC derived from ACS patients. After 48 hour of culture, PBMC conditioned media were collected and analyzed for the release of angiogenic cytokines. Cytokine levels were analyzed in relation to the ability of the PBMC ACS patient samples to generate EPC/ECFC and/or CFU-EC colonies: EPC/ECFC^neg^ vs EPC/ECFC^pos^ gray box-plots) or CFU-EC^neg^ vs CFU-EC^pos^ (white box-plots). Horizontal bars are median, upper and lower edges of box are 75th and 25th percentiles, lines extending from box are 10th and 90th percentiles. Asterisk, p<0.01.

We have next investigated the potential correlation between the occurrence of CFU-EC and/or EPC/ECFC and the release of pro-angiogenic cytokines by the same patient PBMC. For this purpose, patient PBMC conditioned media were collected and analyzed for the release of angiogenic cytokines, such as HB-EGF, KGF, TPO, PDGF-AA, VEGFR-1, VEGFR-2. As shown in **Figure 2**, cytokine levels were analyzed by subdividing and comparing the ACS patient PBMC samples on the basis of their ability to generate CFU-EC and/or EPC/ECFC colonies: *i*) CFU-EC^pos^ vs CFU-EC^neg^; *ii*) EPC/ECFC^pos^ vs EPC/ECFC^neg^. The investigated cytokines were expressed by the PBMC at variable levels, with HB-EGF and KGF either under or very close to the detection limit of the assay (3.7 and 1.95 pg/ml, respectively) in most samples and without any significant difference among the PBMC subgroups. On the other hand, TPO, PDGF-AA, VEGFR-1, VEGFR-2 were released at consistent levels by the PBMC samples assessed. Of interest, a significant higher release of PDGF-AA (p<0.01) characterized the EPC/ECFC^pos^ PBMC, with respect to the other subgroups (**Figure 2**), suggesting a correlation between the release of these cytokines and the circulating EPC/ECFC, which was confirmed by Pearson analysis (R = 0.75, p<0.01). No significant correlations were found between the generation of CFU-EC and the levels of the different cytokines tested.

### Identification of optimal culture conditions for the identification and *ex-vivo* expansion of EPC/ECFC

**Figure 3:**
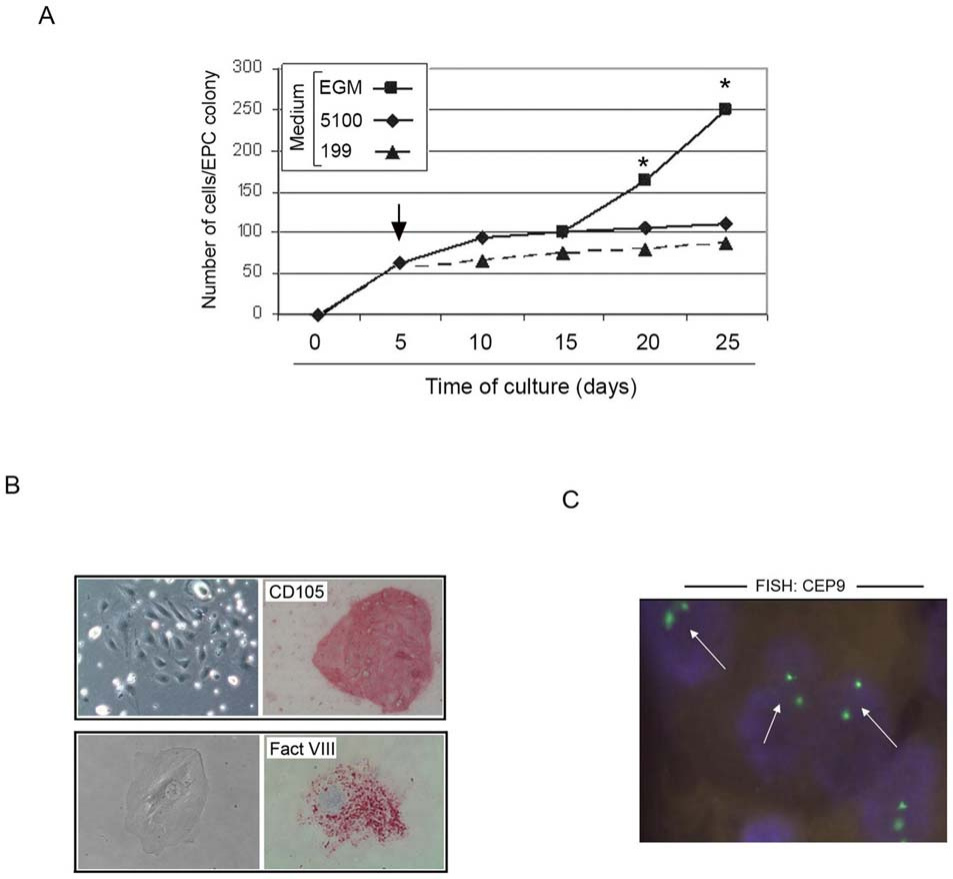
Identification of optimal culture conditions for the *ex-vivo* expansion of ACS PB-derived EPC/ECFC. Primary EPC/ECFC colonies were generated by plating patient PBMC in M^5100^ medium, as detailed in the Method section. In **A**, after the colony identification (at day 5 after plating), medium was change (arrow) and replaced either with fresh M^5100^, or M^EGM^ or M^199^ and the development of the colonies was monitored over the time. The growth kinetics of a representative experiment out of five independent experiments is shown. At each indicated time point, the mean cell number/ECFC was determined by two independent operators; standard deviations were below 10% and are not shown. Asterisk, p<0.05. In **B**, immunocytochemical analysis of *in vitro* expanded EPC/ECFC documenting positivity for CD105 antigen (original magnification: 20X) and for the specific endothelial marker Factor VIII (original magnification: 40X). In **C**, FISH analysis performed on *in vitro* expanded EPC/ECFC by using the centromeric enumeration probe CEP9 (white arrows) documenting a normal diploid chromosomal pattern (original magnification: 40X).

For the identification of primary EPC/ECFC, patient PBMC were seeded in three different culture media (as detailed in the Methods). Growth of EPC/ECFC was detected only by using the M^5100^ medium, while and M^EGM^ and in M^199^ were ineffective for this purpose. In order to perform further cell characterizations, we searched for the optimal culture conditions for the *in vitro* expansion of the primary EPC/ECFC, by assessing the change of medium after the initial plating in M^5100^. Indeed, while M^5100^ medium was necessary to obtain primary colonies, reaching a mean number of 102±25 cells/colony after 15 days of culture, a switch of the medium to M^EGM^, which is a medium particularly enriched of angiogenic cytokines, after the colony identification (approximately at day 5 after PBMC plating), significantly (p<0.05) improved the growth kinetics (**Figure 3A**).

Upon *in vitro* expansion, primary EPC/ECFC were characterized by immunohistochemical analysis, showing a uniform positivity for the specific endothelial marker Von Willebrandt factor (Factor VIII), as well as for CD105 (**Figure 3B**) and CD31 (data not shown). As far as the expression pattern of these markers is concerned, differences were noticed about the intensity and the antigens localization. In particular, the expression of the factor VIII appeared as an intense punctate perinuclear staining (**Figure 3B**). On the other hand, the KDR (VEGFR-1) antigen was weakly expressed by all cells and CD106 (V-CAM) is normally expressed by a lower percentage of activated EPC/ECFC (data not shown). CD14 and CD45 resulted negative. In addition, FISH analysis, performed by using centromeric enumeration probes, allowed to demonstrate a normal diploid chromosomal pattern in the *in vitro* expanded EPC/ECFC (**Figure 3C**).

### Immuno-phenotype and subcloning potential of EPC/ECFC

**Figure 4:**
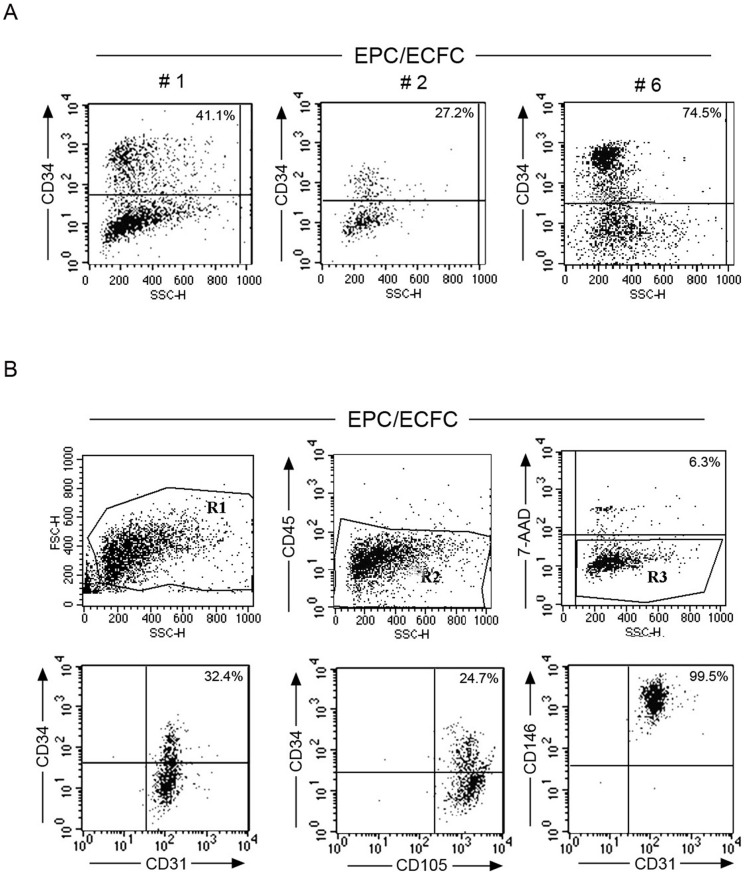
Immunophenotype of EPC/ECFC generated from the PBMC of ACS patients. After *ex-vivo* expansion, primary EPC/ECFC colonies were trypsinized and assessed for their immuno-phenotype by multi-colors flow cytometry. In **A**, the variable expression of the CD34 antigene is documented by 3 independent examples of EPC/ECFC colonies. In **B**, 4-colors flow cytometric analysis of EPC/ECFC cells. A representative example of 7 independent experiments is shown.

**Figure 5:**
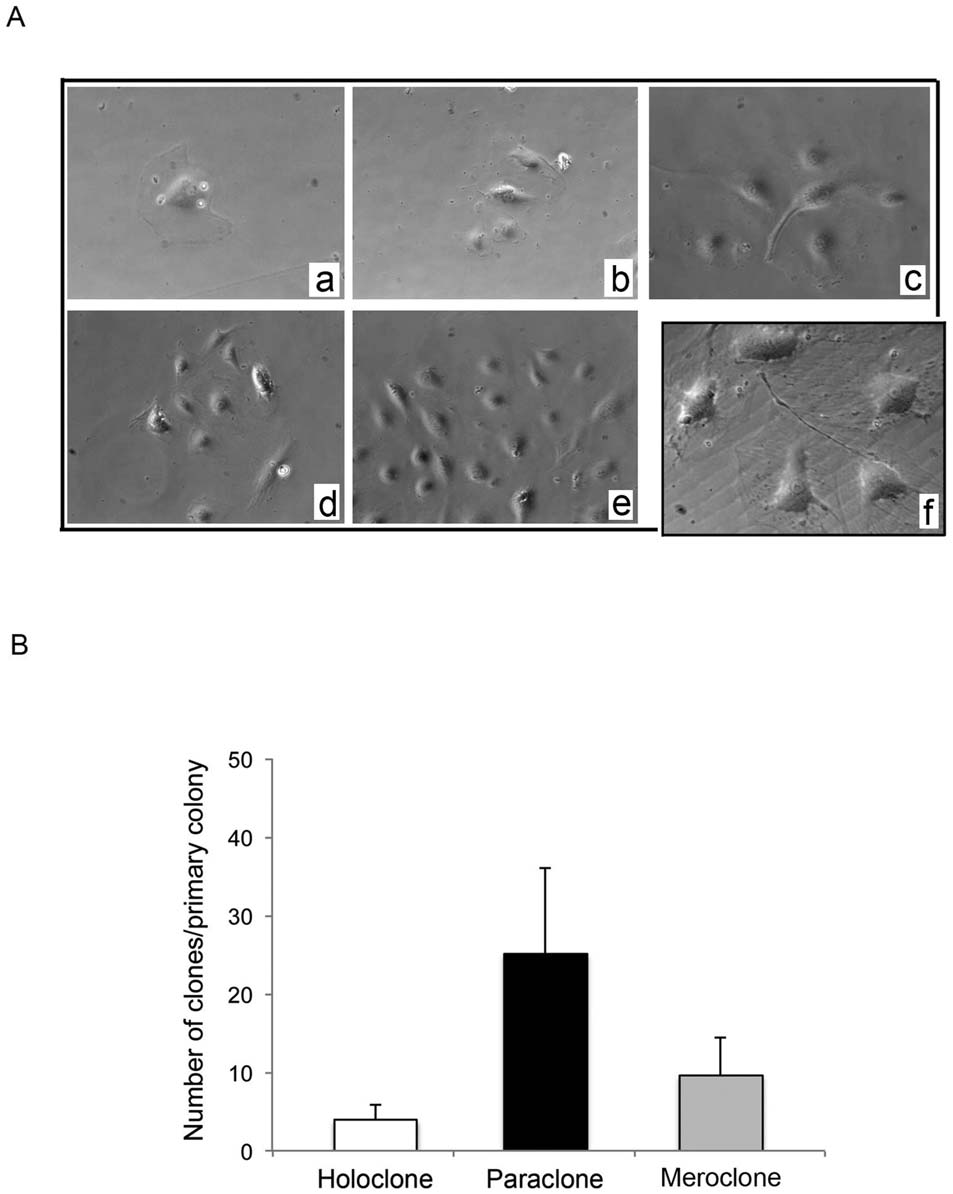
Subcloning potential of EPC/ECFC generated from the PBMC of ACS patients. After *ex-vivo* expansion, primary EPC/ECFC colonies were trypsinized and assessed for clonogenic potential capacity by single cells replating assay. In **A**, single cells derived from EPC/ECPF colonies were seeded in collagen I coated wells and monitored day by day (**a**: day 1; **b**: day 2; **c**: day 3; **e–f**: day 4; **a–e**: original magnification 25X; **f**: original magnification 40X). One representative experiment is shown. In **B**, secondary clones were classified on the basis of their proliferation properties. Data are mean±SD derived from six independent experiments.

After isolation from the ACS PBMC and *ex-vivo* expansion, primary EPC/ECFC colonies were trypsinized and assessed for: *i*) their immuno-phenotype, by multi-colors flow cytometry (**Figure 4**) as well as for *ii*) clonogenic potential capacity, by single cells sub-culturing (**Figure 5**). As documented in **Figure 4A**, EPC/ECFC colonies were characterized by a variable expression of the CD34 antigen, ranging from 20-75% among the different cell samples. Moreover, a 4-colors flow cytometric analysis showed that viable cells from EPC/ECFC colonies were CD45 negative and by gating on cultured CD34^+^/CD45^-^/7-AAD^-^ EPC/ECFC, the expression of CD105, CD31 and CD146 resulted uniformly positive (**Figure 4B**). On the other hand, EPC/ECFC were always negative for CD90, CD117 and CD133, while the expression of CD106 and CD184 was variable (data not shown).

To evaluate the clonogenic potential of EPC/ECFC, a single cell plating (**Figure 5A**) was performed and the resulting clones were assigned to one of the established classes in agreement with the description of Barrandon & Green [28] **:** *i*) large rapidly growing colonies were defined “holoclones”, *ii*) colonies characterized by limited growth were defined “paraclones”, *iii*) colonies showing intermediate features were defined “meroclones”. As result of these analyses, after sub-cloning we calculated a mean clonogenic output of 38.2±18.5 clones/single primary EPC/ECFC seeded, with a prevalence of paraclones with respect to meraclones and holoclones (**Figure 5B**).

## Discussion

Revascularization of tissue following a cardiac infarct is one of the aims of conventional therapy and EPC have been widely studied as a potential source of cell-based therapy for several cardiovascular disorders [29]–[34]. Although putative EPC have been commonly identified and enumerated by flow cytometry, even though without a standardized immunophenotyic approach, few studies have attempted to relate the *in vitro* isolation and expansion of PB-derived EPC to the immunophenotye of putative circulating EPC, and even less information is available about the clonogenic potential of the different endothelial subpopulations. To date, the potential use of EPC/ECFC for cell therapy purposes, especially in cardiovascular diseases, is unclear and many questions concerning the characteristics of these cells are still unresolved. Considering that it is universally accepted that the progenitor cells are defined by their clonogenic expansion capacity, we have undertook this study in order to better highlight EPC/ECFC immunophenotypic and clonogenic properties in patients affected by cardiovascular diseases, as a first essential step to explore the possibility to use these cells in a clinical autologous setting. Obviously, a suitable criterium to isolate and obtain EPC/ECFC is needed and could enhance and address our knowledge for subsequent studies. In this respect, we were able to demonstrate for the first time that, after the initial peak of circulating EPC/ECFC described within the first 3 hours after in-hospital admission for acute myocardial infarction [9]–[11], these true endothelial progenitors are also present in the PB of patients between 7 and 14 days after the cardiovascular event. On the contrary, the presence of CFU-EC monocytic cultures was frequently observed in ACS patients both at early (3–6 days) and late (7–14 days) time points after ACS.

It should be noticed that, as far as the flow cytometric analysis of whole fresh samples is concerned, we failed to predict the presence of circulating EPC on the basis of a described multi-parametric flow cytometric approach. Indeed, the percentage of KDR^+^CD133^+^CD34^+^CD45^-^ cells was very low and similar in all groups of ACS patients. On the other hand, we provided evidence for the first time of the presence of PB-derived EPC/ECFC confined to a late interval (between 7 to 14 days) after the acute event and of their different clonogenic potential. Of note, the presence of primary EPC/ECFC was positively correlated to the release of PDGF-AA in PBMC-derived culture medium supernatant. In this respect, PDGF isoforms are recognized as potent mitogens for connective tissue cells, including dermal fibroblasts, glial cells, arterial smooth muscle cells and some epithelial and endothelial cells. The PDGF-AA isoform is preferentially secreted by fibroblasts, vascular smooth muscle cells, osteoblasts [35], as well as by different malignant cells [36]. Therefore, although it has been proposed that PDGF-AA plays a key role in bone regeneration [35], our current data suggest that the pro-angiogenic activity of PDGF-AA [37] might be essential to recruit EPC/ECFC in the general circulation.

By using media specific for endothelial cell growth, EPC/ECFC, but not CFU-EC, displayed *in vitro* expansion capacity. In addition, FISH analysis conducted with different centromeric probes, revealed encouraging results on EPC/ECFC normal chromosomal numerical pattern thus supporting the idea that these cells may be suitable for clinical applications in regenerative medicine. Taking advantage of the nomenclature recently proposed for a different cell type by Barrandon e Green [28], we have isolated and classified the progeny of EPC/ECFC, distinguishing these clones in holoclones, meroclones and paraclones on the basis of their decreasing *in vitro* expansion capacity. Our results showed that primary EPC/ECFC are functional active since they are clonogenic, giving rise to a different progeny with distinct clonogenic potential while monocytic CFU-EC are not. After subcloning only a part of the primary EPC/ECFC colony give rise to a progeny, and this progeny could be mostly defined as meroclones (containing a mixture of cells of different growth potential) and paraclones. The low frequency of holoclones with the highest growth potential means that only few cells of the primary EPC/ECFC retain the greatest clonogenic expansion capacity of the parental colonies and likely contain endothelial stem cells. These data are also reinforced by the immunophenotypic findings after in vitro culture since a variable number of ECFCs expressing CD34 stem cell marker was found as in the primary ECFCs colonies well as in the progeny. The functional diversity in an apparent homogenous EPC/ECFC cultures has implications for the design of research studies using isolated endothelial progenitor cells with the greatest clonogenic expansion capacity to employ for tissue engineering.

## Supporting Information

Table S1 **Characteristics of the study participants**
(DOC)
