# Supplementary material for: In Vitro Characterization of Circulating Endothelial Progenitor Cells Isolated from Patients with Acute Coronary Syndrome
Source: PLoS One. 2013 Feb 11;8(2):e56377. doi: 10.1371/journal.pone.0056377 (PMC3569417; doi:10.1371/journal.pone.0056377)
Supplement: Table S1 — Characteristics of the study participants (DOC) [file pone.0056377.s001.doc]

**Table S1. Characteristics of the study participants**

| **Variables** | **ACS Patients (n=70)** |
| --- | --- |
| ***General parameters:***  Age (yrs, mean±SD) | 64.5±10.5 |
| Men (%)  Weight (Kg, mean±SD) | 56 (72%)  77.9±12.3 |
| BMI (kg/m^2^, mean±SD)  ***ACS diagnosis:***  NSTEMI (%)  STEMI (%)  UA (%) | 26.9±2  15 (21.4%)  38 (54.2%)  17 (24.4%) |
| ***Biochemical and hematological parameters:*** |  |
| Dyslipidemia (%) | 32 (41.5%) |
| CRP (mg/dl, mean±SD)  WBC (10^3^/μl, mean±SD)  Platelets (10^3^/μl, mean±SD)  Hb (g/dl, mean±SD)  ***Medical history:***  Smoking (%) | 1.8±0.56  8462±2586  237±80.9  13.4±1.1  34 (48 %) |
| Familiarity (%) | 33 (47%) |
| Hypertension (%) | 47 (67%) |
| Diabetes mellitus (%) | 19 (27%) |

BMI: body max index; NSTEMI: non-ST segment elevation myocardial infarction; STEMI: ST-elevation myocardial infarction; UA: unstable angina; CRP: C-reactive protein; WBC: white blood cells; Hb: hemoglobin.
